# A study protocol for a randomised controlled feasibility trial of an intervention to increase activity and reduce sedentary behaviour in people with severe mental illness: Walking fOR Health (WORtH) Study

**DOI:** 10.1186/s40814-021-00938-5

**Published:** 2021-11-15

**Authors:** Suzanne M. McDonough, Sarah C. Howes, Maurice Dillon, Judith McAuley, John Brady, Mary Clarke, Mike Clarke, Emily Lait, Duana McArdle, Tony O’Neill, Iseult Wilson, Ailsa Niven, Julie Williams, Mark A. Tully, Marie H. Murphy, Catherine M. McDonough

**Affiliations:** 1 Centre for Health and Rehabilitation Technologies, School of Health Sciences, Ulster University, Shore Road, Newtownabbey, BT37 0QB UK; 2 School of Physiotherapy, RCSI University of Medicine and Health Sciences, Dublin, Ireland; 3 School of Physiotherapy, University of Otago, Dunedin, New Zealand; 4 Louth Meath Mental Health Services, Midlands Louth Meath Community Healthcare Organisation CHO, Dublin, Ireland; 5 Community Mental Health Team, Northern Health and Social Care Trust, Antrim, UK; 6 Community Mental Health Team, Western Health and Social Care Trust, Omagh, UK; 7 School of Medicine and Medical Sciences, University College Dublin, Dublin, Ireland; 8 School of Medicine, Dentistry and Biomedical Sciences, Queen’s University Belfast, Belfast, UK; 9 Beaumont Hospital, Dublin, Ireland; 10 Centre for Evidence and Social Innovation, Queen’s University Belfast, Belfast, UK; 11 School of Nursing and Midwifery, Queen’s University Belfast, Belfast, UK; 12 Physical Activity for Health Research Centre (PAHRC), Institute for Sport, Physical Education and Health Sciences, University of Edinburgh, Edinburgh, UK; 13 Centre for Implementation Science, Health Service and Population Research Department, Institute of Psychiatry, Psychology and Neuroscience, King’s College London, London, UK; 14 Institute of Mental Health Sciences, School of Health Sciences, Ulster University, Newtownabbey, BT37 0QB UK; 15 Centre for Exercise Medicine, Physical Activity and Health, Sports and Exercise Sciences Research Institute, Ulster University, Jordanstown Campus, Newtownabbey, BT37 0QB UK; 16 Louth Meath Rehabilitation Service, Midlands Louth Meath Community Healthcare Organisation CHO, Dublin, Ireland

**Keywords:** Physical activity, Sedentary behaviour, Behaviour change, Severe mental illness

## Abstract

**Background:**

People with severe mental illness (SMI) are less physically active and more sedentary than healthy controls, contributing to poorer physical health outcomes in this population. There is a need to understand the feasibility and acceptability, and explore the effective components, of health behaviour change interventions targeting physical activity and sedentary behaviour in this population in rural and semi-rural settings.

**Methods:**

This 13-week randomised controlled feasibility trial compares the Walking fOR Health (WORtH) multi-component behaviour change intervention, which includes education, goal-setting and self-monitoring, with a one-off education session. It aims to recruit 60 inactive adults with SMI via three community mental health teams in Ireland and Northern Ireland. Primary outcomes are related to feasibility and acceptability, including recruitment, retention and adherence rates, adverse events and qualitative feedback from participants and clinicians. Secondary outcome measures include self-reported and accelerometer-measured physical activity and sedentary behaviour, anthropometry measures, physical function and mental wellbeing. A mixed-methods process evaluation will be undertaken. This study protocol outlines changes to the study in response to the COVID-19 pandemic.

**Discussion:**

This study will address the challenges and implications of remote delivery of the WORtH intervention due to the COVID-19 pandemic and inform the design of a future definitive randomised controlled trial if it is shown to be feasible.

**Trial registration:**

The trial was registered on clinicaltrials.gov (NCT04134871) on 22 October 2019.

## Background

People with severe mental illness (SMI) experience a higher prevalence of preventable physical health conditions compared to the general population [1]. In particular, people with SMI, such as schizophrenia-spectrum disorders, bipolar disorder and major depressive disorder, have a risk of cardiovascular and metabolic diseases 1.4–2.0 times higher than the general population [2]. Longitudinal data indicates that people with SMI are more likely to have two or more comorbidities, with greater increases in the prevalence of comorbidities over time in people with SMI compared with the general population [1]. Alongside factors including genetic risk and the side effects of anti-psychotic medications, the high prevalence of cardiometabolic-related morbidity and mortality in SMI has been associated with modifiable lifestyle factors, including reduced physical activity and high levels of sedentary behaviour [3].

Systematic review evidence has shown that people with SMI are less physically active and spend more time in sedentary behaviour than healthy controls [4–9]. They complete less moderate to vigorous physical activity than controls [9], with up to 70% not meeting physical activity guidelines [4, 10]. They also spend 8–11 h per day sitting or lying down, which represents more time in sedentary behaviour than healthy controls [4, 5, 7–9]. Higher levels of sedentary behaviour in SMI have been associated with poorer metabolic outcomes [11], while increased physical activity improves cardiometabolic risk [12]. Interventions that support those who are inactive to replace sedentary time with small amounts of physical activity are likely to reduce the incidence and impact of cardiometabolic comorbidities in people with SMI [13–15].

Barriers to being active experienced in the general population, such as time constraints and physical health concerns, are compounded in people with SMI due to their mental health symptoms. People with SMI report physical barriers, such as tiredness, low energy and sedative effects of their medications; psychological barriers, such as stress, depression and amotivation; and social and environmental barriers, such as lack of support and social isolation [16–18]. Given these barriers, and the high levels of inactivity in people with SMI, walking has been recommended as one of the simplest ways of increasing physical activity [19]. Systematic review evidence shows small, short-term effects of walking on weight reduction (*n* = 10 trials, 339 participants) [20], and recent feasibility evidence by members of our team [21] showed that a walking-based intervention to increase physical activity and reduce sedentary behaviour in adults with SMI was feasible and acceptable, with positive findings in recruitment, retention, adherence and participant feedback and preliminary evidence for an increase in physical activity levels and reduction in sedentary behaviour in the intervention compared to the control group.

Although the study by Williams et al. [21] contributes to the developing knowledge base on walking interventions in people with SMI, it was conducted in a densely populated urban setting. Environmental design features of large cities, such as the walkability of streets, proximity of destinations and density of shops and services close to the home, strongly influence the likelihood of walking [22]. Conversely, residents of rural and semi-rural areas have to overcome additional unique barriers to engage in regular walking, such as a greater dispersion of housing, infrastructure and services leading to increased car-dependency and concerns about personal safety, for example walking on unlit rural roads without footpaths [23]. As such, an urban-based intervention may not be directly transferable.

This randomised controlled feasibility study aims to assess the feasibility and acceptability of a multi-component behaviour change intervention and explore its efficacy in improving physical activity and sedentary behaviours in adults with SMI living in rural and semi-rural environments. It also aims to explore effective intervention components to support health behaviour change in this study population. Findings will be used to optimise design of a main fully powered trial.

## Aims and objectives

### Aim

To test the feasibility of a multi-component behaviour change intervention aimed at increasing physical activity and reducing sedentary behaviour compared with a one-off education session in people with SMI living in rural and semi-rural locations.

### Objectives

i. To determine the recruitment, retention and adherence rates in both trial arms and explore reasons for these rates.
ii. To determine the acceptability of the intervention in terms of the incidence of adverse events and level of overall satisfaction in both groups.
iii. To estimate variability in clinical markers to inform the design of future effectiveness studies (calculate effect sizes for change in physical activity, sedentary behaviour and cardiometabolic risk factors, including BMI and waist circumference).
iv. To conduct a process evaluation according to MRC guidance [24] to explore potential mediators of behaviour change (motivation to exercise and psychological needs satisfaction), to determine requirements for clinical staff to deliver the intervention, and service users views of the intervention.

## Methods

This section describes the planned methodology; changes made due to the COVID-19 pandemic are summarised later in Table 2.

### Study design

**Fig. 1:**
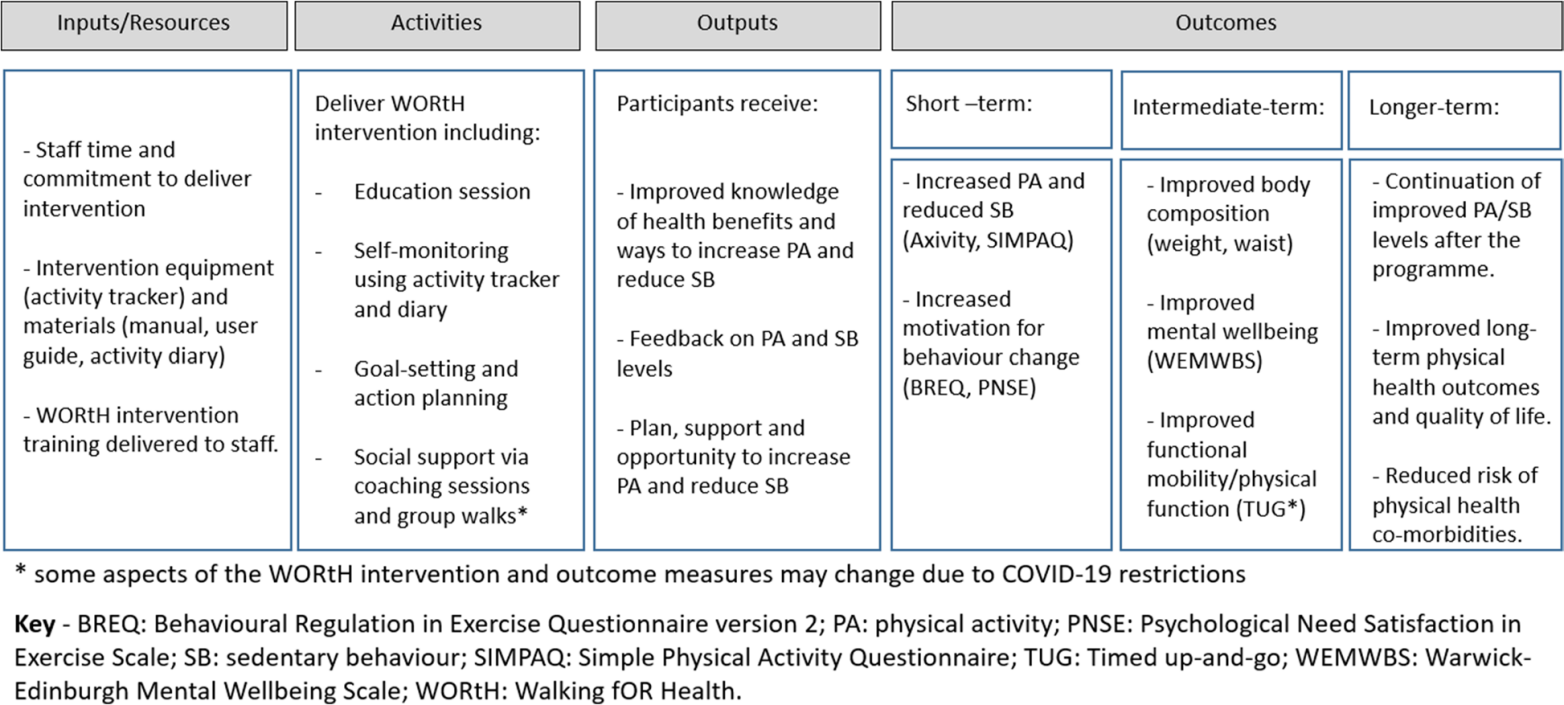
A logic model of the proposed effect of the WORtH intervention on health in individuals with SMI

This feasibility study is a 13-week randomised controlled trial comparing the Walking fOR Health (WORtH) intervention, a group-based intervention including education, goal-setting, self-monitoring, and group walks, with a control consisting of a single education session during which participants receive written and verbal information on the benefits of being more active. A logic model detailing how the WORtH intervention may achieve its proposed outcome is presented in Fig. 1.

### Setting

The intervention will take place within the mental health service in two health care trusts in Northern Ireland (NI) and one in the Republic of Ireland (RoI).

### Approval

Ethical approval for the trial has been obtained from the Office for Research and Ethics Committees NI (IRAS ID: 250401) and the Health and Social Care Executive Dublin North East Research Ethics Committee in RoI. Local research governance approval was obtained from the relevant health care trusts. The trial has been registered on clinicaltrials.gov (NCT04134871).

### Study population

This trial aims to recruit 60 participants: 30 in the intervention group and 30 in the control group. A formal sample size calculation was not used for this feasibility study; it is anticipated that this sample size will provide sufficient information on the study objectives to inform a future main trial. The inclusion criteria will be adult participants (male or female; aged ≥ 18 years) with a diagnosis of any SMI (schizophrenia, psychosis, bipolar disorder and major depression), not meeting the national physical activity guidelines. A validated questionnaire, the General Practice Physical Activity Questionnaire (GPPAQ), will be used to screen physical activity levels [25]. Exclusion criteria will be those with significant movement impairment, those identified as ‘Active’ using the GPPAQ screening tool and those unable to understand English or who lack comprehension to understand the purpose of the study and give written informed consent. The revised Physical Activity Readiness Questionnaire (PAR-Q) [26] will be used to identify any health-related risks of exercising that would require a participant to consult their general practitioner prior to changing their physical activity levels.

### Recruitment

Recruitment to the trial will be via mental health teams in NI and ROI and will involve a number of strategies. The research team will work closely with the multidisciplinary mental health team clinicians and the Northern Ireland Clinical Research Network Mental Health group, where available, to identify service users who meet the eligibility criteria. Recruitment will be via screening clinic lists, referral from existing services, such as groups, day-care services and residential facilities, and through posters displayed in communal and waiting areas. Service users who meet the eligibility criteria will be given information about the study, either at a clinic visit or via post. Those who express an interest in taking part will be invited to meet with the researcher to complete the GPPAQ and PAR-Q and be given the opportunity to ask any questions they have about the study. Following this, if interested in participating in the study they will be asked to provide written consent.

### Randomisation

Cluster randomisation will be used given that some participants may be recruited from services such as groups, day centre or residential facilities. Each cluster will have a 1:1 probability of being allocated to intervention or control. The randomisation will be done by a researcher independent of the study and blind to the identity of the participants. A site will be ready for randomisation when all participants have consented and completed baseline assessment. Random allocation to intervention or control will be done when a group of sites are ready for randomisation.

### Intervention

Participants assigned to the WORtH intervention group will attend an initial educational group session where they will meet their clinician-coach, a clinician from the mental health team who has been trained to deliver the WORtH intervention. The content of the group education session will introduce the benefits of increasing their physical activity and reducing their sedentary behaviour, along with strategies to help them to move more and sit less in their daily routines. During the session, participants will be given a physical activity monitor (Mi Band 3, Xiaomi Corporation, Hong Kong) to self-monitor their daily steps throughout the intervention and an activity diary where they will record their physical activity and sedentary behaviour goals, complete their action plans and self-monitor by recording daily goal attainment.

Participants will be invited to attend a weekly group walk led by a clinician-coach; this will provide an element of social support. Additionally, they will meet with their assigned clinician-coach every 2 weeks. During coaching sessions, the participant will be supported to set move more (physical activity) and sit less (sedentary behaviour) goals which will be reviewed and progressed during the programme. Participants will be supported to complete an action plan of when, where and how they plan to meet their goals including strategies to overcome anticipated barriers discussed during the session.

**Fig. 2:**
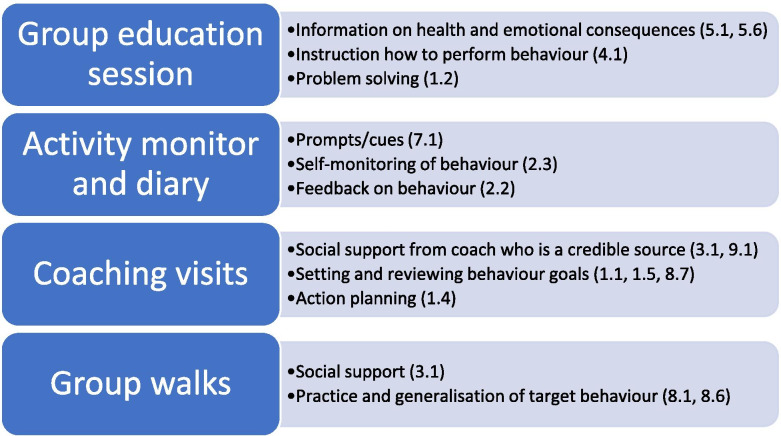
Summary of behaviour change techniques included in the intervention

The Behaviour Change Technique Taxonomy version 1 (BCTTv1) [27] provides a standardised and well-defined taxonomy of active components of behaviour change that can be used in the design and evaluation of interventions [28]. This approach has been used by our team to develop and define a number of physical activity and sedentary behaviour interventions [29–32] and helped to inform the behaviour change techniques (BCTs) to embed within the WORtH intervention to promote increased physical activity and reduced sedentary behaviour in people with SMI. WORtH intervention components were mapped to the BCTTv1 [27] independently by two authors (SH and AN) to identify the BCTs targeting increased physical activity and reduced sedentary behaviour in people with SMI. A summary of the core BCTs embedded within the WORtH intervention is presented in Fig. 2.

### Control

Participants assigned to the control group will be invited to attend a single one-to-one information session where they will be given an A5 leaflet briefly outlining the benefits of being more active and reducing sedentary behaviour and advice on how to do so. During the session, the researcher will read through the leaflet with the participant and give them the opportunity to ask questions. The researcher will complete a pro-forma with details of the duration of the session and a checklist of information to be covered in the session. At the end of the session, they will be informed of the date of end of study assessment. Immediately after the session, the researcher will make a handwritten note of the conversation and comments.

### Outcomes

#### Primary outcome

The feasibility and acceptability of the intervention will be evaluated in terms of recruitment, retention and adherence rates to the trial. Where available, reasons for these rates will be recorded (study objective I). A record will be kept of all information on instances of adverse events, including mental and physical problems and any reports of difficulty with intervention components (study objective II).

#### Secondary outcomes

**Fig. 3:**
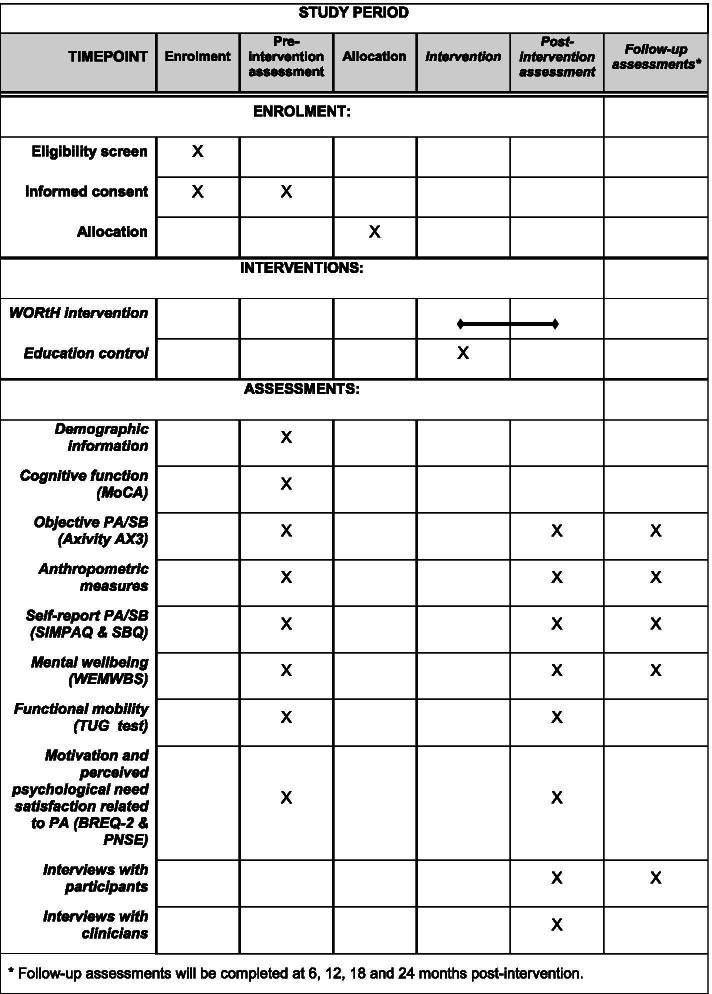
SPIRIT schedule of enrolment, intervention and assessment

The following outcomes will be measured at baseline and post-intervention, with a subset included at 6, 12, 18 and 24 months follow-up (study objective III) as summarised in Fig. 3:

- Objective sedentary and physical activity time: All participants will be asked to wear a wrist-worn triaxial accelerometer (Axivity AX3, Open Lab, Newcastle) for at least 4 days. The accelerometer will record how many minutes per day each participant is sedentary and engages in light, moderate and vigorous physical activities. This study will explore the change in average minutes of sedentary behaviour and physical activity before and after the intervention (study objective III).
- Anthropometric measures: Body mass index will be measured in line with the International Diabetes Federation criteria [33], including height, weight and waist circumference.
- Self-report of physical activity and sedentary behaviour: The Simple Physical Activity Questionnaire (SIMPAQ) will be used to capture self-reported physical activity [34]. The SIMPAQ includes five items asking about estimated time in bed, structured exercise participation and incidental or non-structured physical activity over the previous 7 days. The Sedentary Behaviour Questionnaire (SBQ) will be used to measure sedentary behaviour and has been shown to have good reliability [35].
- Mental well-being: Participants will be asked to complete the Warwick-Edinburgh Mental Wellbeing Scale (WEMWBS), a 14-item self-report measure, to assess well-being [36]. Respondents rate their experience regarding each statement over the last 2 weeks. Each item is scored using a 5-point Likert scale ranging from 1 (none of the time) to 5 (all of the time), with the total score ranging from 14 (low well-being) to 70.
- Functional mobility: Participants will be asked to complete a Timed Up-and-Go (TUG) test [37]. The TUG test requires participants to stand up from a chair, walk 3 m, turn around, walk back and sit down again. The time taken is measured in seconds, and scores represent functional mobility, with higher scores indicating increasing mobility difficulties. Times longer than 13.5 s are predictive of falls in the general older adult population.
- Motivation to engage in physical activity: Participants will be asked to complete the Behavioural Regulation in Exercise Questionnaire-2 (BREQ-2) which is a 19-item interviewer-administered questionnaire designed to consider an individual’s motivation towards exercise [38]. The BREQ-2 has been validated in people with schizophrenia [39]. They will also be asked to complete the Psychological Needs Satisfaction and Exercise Scale (PNSE), an 18-item questionnaire that measures perceived competence, autonomy and relatedness experienced in exercise contexts [40].

At baseline, socio-demographic information will be collected, including age, gender, ethnicity, living arrangements, pain level, smoking status, psychiatric diagnosis and current medications. Additionally, at baseline, all participants complete the Montreal Cognitive Assessment (MoCA) [41], a 30-item screening tool that is validated in SMI [42] and examines cognitive domains including executive functioning, attention and verbal fluency. Scores < 26 are suggestive of cognitive impairment. This data will be used to understand the implications of cognitive impairment on intervention delivery and completion of outcome assessments.

For the post-intervention assessment, attempts will be made to follow all participants, including those who discontinue the intervention early. Follow-up assessments at 6, 12, 18 and 24 months will be optional and will include the outcomes summarised in Fig. 3. Additionally, information on participants’ continuation with intervention components after the end of the programme will be collected.

Process evaluation (study objective IV): In addition to the outcome assessment, a qualitative process evaluation will be conducted to explore participants’ experience of the intervention, factors influencing engagement with the intervention and intervention changes required. Qualitative data from semi-structured interviews will be considered alongside the BREQ-2 and PNSE measures to consider how motivational constructs may mediate the effects of the intervention on behaviour change.

The process evaluation will also explore how the clinicians delivering the coaching intervention experienced this; qualitative data collection will explore their ability to deliver the instructions as planned, including facilitators and barriers to the delivery, the sufficiency of the training and the support from the research team. Members of the wider mental health care team who are involved in supporting participants with aspects of the intervention will also be invited to a focus group, dyad or interview (depending on the availability of individuals) to explore the level of support required by participants and the challenges and facilitators to complete the intervention from their perspective.

To explore the extent to which the intervention is delivered as planned, clinician-coaches will be asked to audio-record coaching sessions. Participants will be asked to consent to audio-recording of their coaching sessions. The audio-recordings will be reviewed alongside notes made in the study record forms by clinician-coaches documenting content of coaching session to assess delivery of the active components of the intervention against a study-specific pre-defined checklist based on the Borelli checklist [43, 44].

### Data analysis

Statistical analysis will be performed using SPSS software. The data will be checked for normality, then appropriate descriptive analyses will be used to summarise participant characteristics and outcomes. Feasibility, the primary outcome of this study, will be evaluated by calculating the percentage of people approached who participate in the intervention (recruitment) and the percentage who complete the intervention (retention and adherence).

As this is a feasibility study, significance tests will not be performed on secondary outcomes, such as change in anthropometric measures, physical activity or sedentary time. Intervention effects will be represented by point estimates and their standard deviations. Point estimates will be calculated by subtracting unadjusted mean data at each post-intervention timepoint from the mean data at the baseline timepoint. These will be used along with their standard deviations to estimate a sample size required for a definitive trial, if appropriate [45].

Qualitative data from interviews, dyads and focus groups will be transcribed verbatim, and interpretation, synthesis and data reduction will be undertaken independently by two members of the research team to identify relevant themes. Qualitative findings will be used alongside quantitative outcomes related to process evaluation (BREQ-2 and PNSE) to understand how the intervention was experienced by participants and any changes that they think would improve the intervention.

The traffic light system [46] will be used to guide progression to a main trial, as recommended in recent best practice, and will use the worst performing of the four elements in Table 1 to select which of the following actions to take:

**Table 1:** Criteria for progression to main trial

	Red	Amber	Green
**Recruitment**			
**% of target number of participants recruited (target: 60; 12 month recruitment period)**	< 70%	70–99%	100%
**Adherence**			
**% of recruited participants who adhere to their allocated intervention**	< 65%	65–84%	85–100%
**Retention**			
**% of recruited patients with follow-up data**	< 65%	65–84%	85–100%
**Signal of efficacy**			
**Results for primary clinical outcome and safety**	CI for effect estimate for primary clinical outcome that does not include a clinically important difference or evidence of significant harm	CI for effect estimate for primary outcome that is mostly negative or evidence of potential harm	CI for effect estimate for primary outcome that is mostly positive and no evidence of potential harm

**Green**: progress to main trial with a review of screening logs and protocol to address any barriers to recruitment, adherence or retention.

**Amber**: progress to main trial after discussion with Trial Steering Committee/Trial Management Group with a full review of screening logs and protocol deviations to implement solutions to barriers to recruitment, adherence or retention, and (if relevant) a review of feedback from participants and clinicians, and (if relevant) reconsideration of the intervention.

**Red**: probably not progress to main trial without substantial changes.

### Implications of COVID-19 on study delivery

This study is being rolled out during the COVID-19 global pandemic. Therefore, current public health restrictions in place relating to COVID-19 will be followed, aiming to reduce in-person contact with participants to reduce the risk of transmission for patients and staff, where possible (Table 2). Recruitment to the study was open both pre- and post-suspension due to COVID-19; as such, participants recruited to the trial pre-suspension were cluster-randomised in a 1:1 ratio and received an in-person intervention, while participants recruited post-suspension are individually randomised in a 2:1 ratio and receive remote delivery of the intervention as outlined in Table 2.

**Table 2:** Proposed protocol changes in response to COVID-19

Study activity	Measures taken
Recruitment	Study information will be shared via email, where possible, or post. Screening visit will be completed via telephone. Consent form will be completed and returned by post or email, where possible. Participants can identify a carer to support them. Carer consent will be obtained. Rolling recruitment will take place as there will be no group component to the intervention and to reduce clinician burden when delivering the intervention.
Randomisation	Participants will be randomised at individual level (due to practicalities of rolling recruitment, e.g. to ensure allocation concealment as participants from the same residential setting may enrol at different times) to either intervention or control group in a 2:1 ratio after completion of the baseline assessment. The allocation ratio was changed to ensure sufficient number of participants are exposed to the intervention and optimal information on intervention delivery can be collected, given that there may be recruitment challenges with remote recruitment and delivery. With 2:1 allocation ratio, two-thirds of participants enrolled will be randomised to the intervention group. Allocations will be undertaken using a permuted block design with unequal block sizes (of 3, 6, 9). A researcher independent of the study will generate the randomisation sequence and allocate the participant after baseline data collection. The randomisation sequence will be concealed from members of the research team involved in participant management to prevent biased allocation [47, 48].
Intervention delivery	Education session (both intervention and control groups) will be delivered remotely via telephone or video conference depending on technology accessible to the individual. Study materials will be posted to participants. Instructions will be sent via email or post and the researcher will provide additional instruction via telephone or video call. Fortnightly coaching calls will be delivered remotely by telephone or video call. Delivery of the weekly group walk will depend on current guidance regarding physical distancing.
Outcome assessment	Participants will be given the option to complete questionnaires via email, post, telephone or video call. This will include using alternative versions of tools that are suitable for remote delivery, where required i.e. using the Blind-MoCA, which omits requirement of pencil and paper or visual stimulus, and is suitable for telephone administration of the test [49]. Accelerometers will be posted to participants with instructions and the researcher will provide additional instruction via telephone or video call. Anthropometric measures will be collected from patient files, where available. Exploration of remote methods of collecting data related to functional mobility, i.e. replacing the Timed-Up-and-Go test with a remote Sit-to-Stand test [50]. Interviews with participants, clinicians and carers will be carried out by telephone or video call.

## Discussion

This randomised controlled feasibility study will evaluate the feasibility and acceptability of a multi-component behaviour change intervention to increase physical activity and reduce sedentary behaviour of adults with SMI living in rural and semi-rural environments.

Additionally, this study will address the challenges and implications of remote delivery of the WORtH intervention due to the COVID-19 pandemic. There is limited evidence on the feasibility and acceptability of remotely delivered interventions for the management of SMI [51, 52], and we are not aware of any remotely delivered interventions aimed at improving physical activity and sedentary behaviour in this population. The findings will inform the design of a future definitive randomised controlled trial if it is shown to be feasible.

## Trial status

This trial is ongoing and open to recruitment.

## Data Availability

Data and materials can be requested from the corresponding author.

## Abbreviations

BCTTv1: Behaviour Change Techniques Taxonomy version 1
BREQ-2: Behavioural Regulation in Exercise Questionnaire version 2
GPPAQ: General Practice Physical Activity Questionnaire
MoCA: Montreal Cognitive Assessment
NI: Northern Ireland
PAR-Q: Physical Activity Readiness Questionnaire
PNSE: Psychological Need Satisfaction in Exercise Scale
RoI: Republic of Ireland
SBQ: Sedentary Behaviour Questionnaire
SIMPAQ: Simple Physical Activity Questionnaire
SMI: severe mental health difficulties
TUG: Timed Up-and-Go
WEMWBS: Warwick-Edinburgh Mental Wellbeing Scale
WORtH: Walking fOR Health
